# A Systematic Review and Recommendations Around Frameworks for Evaluating Scientific Validity in Nutritional Genomics

**DOI:** 10.3389/fnut.2021.789215

**Published:** 2021-12-14

**Authors:** Justine Keathley, Véronique Garneau, Daniela Zavala-Mora, Robyn R. Heister, Ellie Gauthier, Josiane Morin-Bernier, Robert Green, Marie-Claude Vohl

**Affiliations:** ^1^Centre Nutrition, Santé et Société (NUTRISS), Institut sur la Nutrition et les Aliments Fonctionnels (INAF), Université Laval, Québec City, QC, Canada; ^2^School of Nutrition, Université Laval, Québec City, QC, Canada; ^3^Mass General Brigham, Boston, MA, United States; ^4^Ariadne Labs, Boston, MA, United States; ^5^Harvard Medical School, Boston, MA, United States; ^6^The Broad Institute, Boston, MA, United States; ^7^Library, Université Laval, Québec City, QC, Canada; ^8^Human Longevity, Inc., San Diego, CA, United States

**Keywords:** nutrition, genetics, nutrigenetics/nutrigenomics, nutritional genomics, systematic review, clinical practice, scientific validity, frameworks

## Abstract

**Background:** There is a significant lack of consistency used to determine the scientific validity of nutrigenetic research. The aims of this study were to examine existing frameworks used for determining scientific validity in nutrition and/or genetics and to determine which framework would be most appropriate to evaluate scientific validity in nutrigenetics in the future.

**Methods:** A systematic review (PROSPERO registration: CRD42021261948) was conducted up until July 2021 using Medline, Embase, and Web of Science, with articles screened in duplicate. Gray literature searches were also conducted (June-July 2021), and reference lists of two relevant review articles were screened. Included articles provided the complete methods for a framework that has been used to evaluate scientific validity in nutrition and/or genetics. Articles were excluded if they provided a framework for evaluating health services/systems more broadly. Citing articles of the included articles were then screened in Google Scholar to determine if the framework had been used in nutrition or genetics, or both; frameworks that had not were excluded. Summary tables were piloted in duplicate and revised accordingly prior to synthesizing all included articles. Frameworks were critically appraised for their applicability to nutrigenetic scientific validity assessment using a predetermined categorization matrix, which included key factors deemed important by an expert panel for assessing scientific validity in nutrigenetics.

**Results:** Upon screening 3,931 articles, a total of 49 articles representing 41 total frameworks, were included in the final analysis (19 used in genetics, 9 used in nutrition, and 13 used in both). Factors deemed important for evaluating nutrigenetic evidence related to study design and quality, generalizability, directness, consistency, precision, confounding, effect size, biological plausibility, publication/funding bias, allele and nutrient dose-response, and summary levels of evidence. Frameworks varied in the components of their scientific validity assessment, with most assessing study quality. Consideration of biological plausibility was more common in frameworks used in genetics. Dose-response effects were rarely considered. Two included frameworks incorporated all but one predetermined key factor important for nutrigenetic scientific validity assessment.

**Discussion/Conclusions:** A single existing framework was highlighted as optimal for the rigorous evaluation of scientific validity in nutritional genomics, and minor modifications are proposed to strengthen it further.

**Systematic Review Registration:** https://www.crd.york.ac.uk/prospero/display_record.php?RecordID=261948, PROSPERO [CRD42021261948].

## Background

It has been suggested for decades that optimal strategies for managing health and disease should be personalized. In 1903, Dr. William Osler stated that “variability is the law of life and no two individuals react alike and behave alike under the abnormal conditions which we know as disease. The good [healthcare provider] treats the disease, the great one treats the patient (1).” In modern times, we are experiencing a new level of personalized healthcare, with the consideration of genetic variation for treating, managing and preventing various diseases and conditions. In the field of nutrition, the use of genetic information to personalize dietary recommendations is a potentially powerful tool emerging as a result of the developing field of nutritional genomics. This is a field that explores the interplay between genetics and nutrition, and their subsequent impact on health-related outcomes (2). While this science is applied to clinical practice and more broad guidance for clinicians has been published (3, 4), there is considerable debate about whether the use of nutrigenetic tests have sufficient scientific validity to be supported (5, 6). In order to resolve this debate, there is a clear need to determine the quality of evidence (scientific validity) of the various nutrigenetic tests and their clinical claims. In addition to scientific validity, various factors should be considered when determining the suitability of a (nutri)genetic test for clinical practice. Analytic validity, clinical utility, ethical/legal implications, and risk vs. benefit are some of these factors; these are considered in the development of comprehensive clinical practice guidelines (CPGs) (7, 8). Scientific validity is one key factor evaluated prior to developing CPGs, and is considered a component of clinical validity, alongside clinical test performance (9). Scientific validity in genetics is about determining whether a gene-disease association is substantiated in the scientific literature (9). Assessing the strength and quality of evidence is a key component of determining scientific validity.

Several methods exist for evaluating the scientific validity in the fields of nutrition (10–13) and genetics (14–18) individually. However, nutritional genomics has unique considerations; as such, guidelines for evaluating genetic research, or for evaluating nutrition research are likely insufficient for properly evaluating the distinctive field of nutrigenetics. This poses concerns given the importance of selecting frameworks for evaluating scientific validity that match the research question and the type of evidence available (19).

Therefore, the aim of this study was to first provide a systematic overview of evidence evaluation frameworks used to assess scientific validity in the fields of both nutrition and genetics. We further aimed to determine if a framework exists that is appropriate for evaluating scientific validity in nutritional genomics. Lastly, we aimed to propose a framework (either an existing framework, modified version or novel framework) for use specifically in determining scientific validity in nutritional genomics. The proposed framework is intended to stimulate discussions in the scientific community and form the foundation for future refinement.

## Methods

This systematic review was registered with PROSPERO (CRD42021261948) and the protocol can be accessed at https://www.crd.york.ac.uk/prospero/display_record.php?RecordID=261948. The review process was guided by previously established methods for conducting rigorous systematic reviews (20, 21).

### Literature Search and Inclusion Criteria

**Figure 1:**
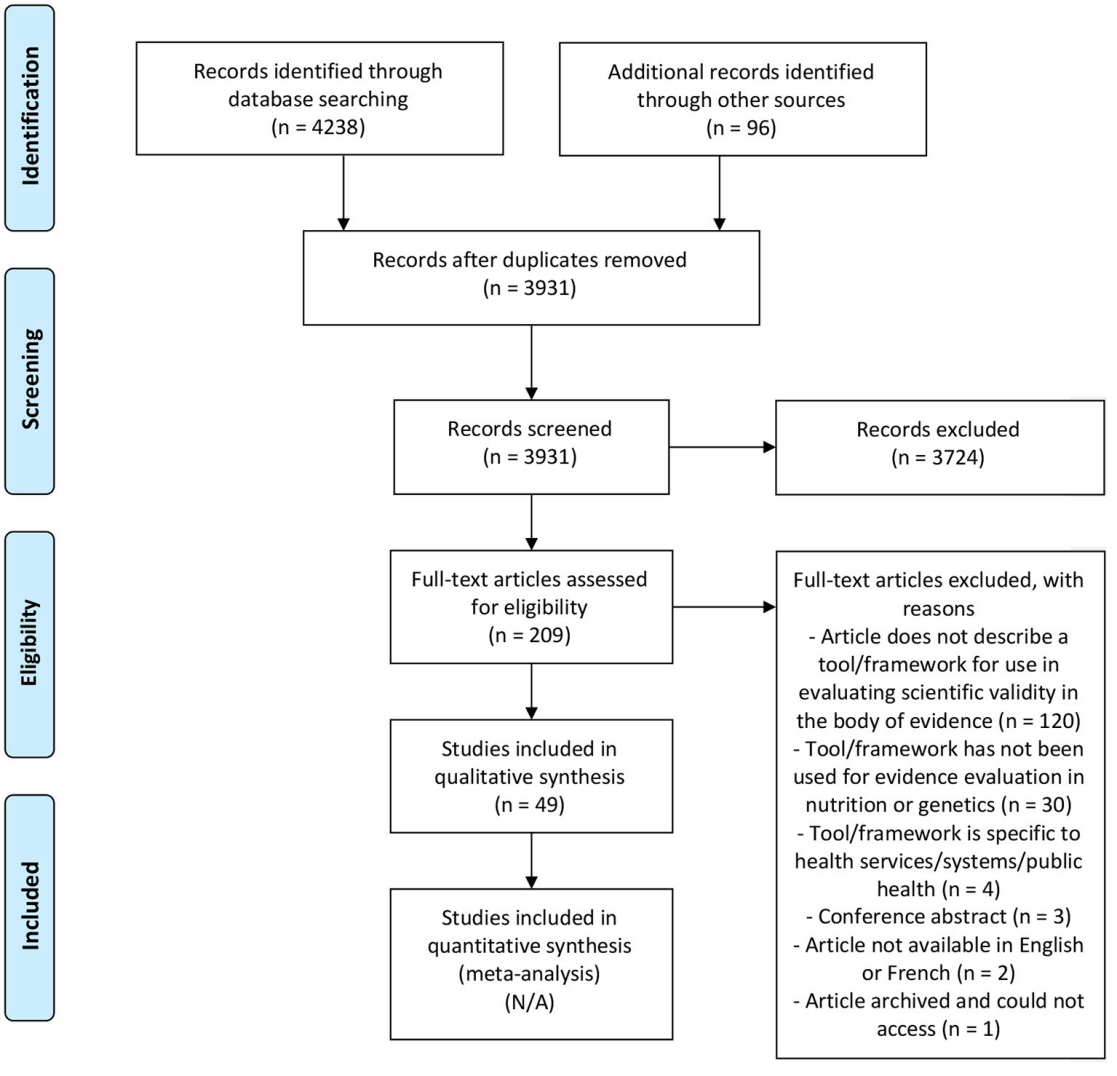
PRISMA 2009 flow diagram (22).

First, a systematic search was undertaken by two investigators using three search engines: Embase, Web of Science, and Medline. The search terms are outlined in Supplementary Table 1, and databases were searched up until July 1, 2021. The PRISMA flow diagram (Figure 1) was used to guide the search strategy, moving from abstract to full-text screening (22). Article screening and selection were completed independently by two authors (JK and VG). First, titles and abstracts were screened in duplicate using Covidence software in which articles that clearly did not meet the eligibility criteria were excluded. Next, full-text articles were screened and the final list of included articles was developed. Reference lists of two relevant articles were also reviewed to search for eligible studies (18, 19). During full-text screening, JK and VG further noted any potentially relevant frameworks mentioned in the full-texts and additionally reviewed these to determine if they met the inclusion criteria. If an article met all inclusion criteria, cited articles were then searched using Google Scholar, to determine if the framework had been used for evaluating scientific validity of the body of evidence in a topic specific to nutrition and/or genetics. An additional gray literature search was conducted in June-July 2021 using the Google search engine using various combinations of key terms: evaluating; evaluation; evidence-based; evidence synthesis; scientific validity; nutrition; genetics; genomics; medicine; tool; approach; guide; manual; method; framework; levels of evidence. Included articles consisted of those that provided the complete methods for an evidence grading framework used to evaluate the quality of scientific evidence (i.e., scientific validity) in nutrition and/or genetics. Articles related to assessing the quality of genetic tests more broadly (i.e., those that considered various aspects of quality such as clinical utility, clinical validity, analytic validity, etc.) were included with the condition that a component of the methodology related to assessing scientific validity; the article summary then focused exclusively on the scientific validity assessment from the framework. Articles related to evidence evaluation frameworks specific to health services/systems more broadly such as those that related to public health program assessment, but not scientific validity in nutrition and/or genetics, were excluded.

### Data Extraction

Data extraction templates were piloted in duplicate on 10 included articles, with one author completing the remaining data extraction (JK). The final data extraction template included the following headings: first author name and year, framework title, included study designs for evidence evaluation, main scientific validity evaluation components described in the tool, categories for levels of evidence, a brief description of the framework and whether the framework has been used in nutrition and/or genetics. Data was also extracted for completion of the categorization matrix, further described below.

### Categorization Matrix and Appraisal of Frameworks

The included frameworks were then critically appraised for their appropriateness for evaluating the quality of nutrigenetic research. A categorization matrix was used to guide the appraisal process; matrix headings were developed within the context of key factors to consider in determining levels of evidence for nutrigenetic study findings. These factors (matrix headings) were drafted collaboratively by the expert working group (JK, VG, M-CV, RG). The first draft was developed based on a review of the literature on standard items included in evidence evaluation tools, critiques of existing evidence evaluation tools and special considerations for evidence evaluation of nutrigenetic research. Following this, an external panel of experts in nutritional genomics and/or knowledge translation (either in nutrition or genetics) reviewed/revised the draft categorization matrix and provided comments. The external panel is listed in the acknowledgments section. Following revisions, all authors and members of the external panel approved the final categorization matrix.

Each included framework was then added to the categorization matrix, and data were extracted to evaluate the suitability of the framework for evaluating evidence in nutrigenetics. Checkmarks indicated that a factor was considered in the framework, whereas an “X” indicated that the factor was not considered in that particular framework. For example, frameworks that incorporated evidence of biological plausibility (as defined in Table 1) as a factor contributing the strength of the evidence received a checkmark in the categorization matrix whereas those that did not consider evidence of biological plausibility received an X. It should be noted that frameworks with more X's are not necessarily less robust, but rather they were deemed less appropriate for *nutrigenetic* evidence evaluation specifically. Based on the results of the categorization matrix, a framework was recommended, with minor revisions, for use in analyzing the scientific validity of gene-diet interactions/associations.

**Table 1:** Description of factors deemed important to consider for evaluating the body of evidence in nutritional genomics.

**Factor considered in evidence evaluation**	**Description**
Quality assessment for study risk of bias/methodological quality	An evaluation of specific components of study methods and whether or not the study has strong internal validity (e.g., appropriateness of statistical analyses, type of study such as observational or interventional, adherence to intervention, validity and reliability of measurements including dietary assessment methods, etc.)
Different study designs included (with different weighting by design)	Higher quality study designs (e.g., randomized controlled trials) deemed as stronger evidence, and lower quality study designs (e.g., cohort studies) as weaker evidence
Population directness/generalizability	Comparison of populations across different studies to determine the generalizability of the results
Study directness (relatedness)	Comparison of aspects of study design (e.g., SNPs tested, interventions/ exposures, etc.) between studies
Statistical precision	The degree of statistical accuracy (e.g., smaller standard errors are more precise than larger standard errors)
Consistency of study results	Whether or not study results are similar between different studies (replication)
Plausible confounding	For observational research, most/all possible confounding factors have been incorporated in the analyses
Effect size	Measurement of the strength of the relationship between two variables, typically considered to be either small, moderate or large
Publication/funding bias	Selective reporting/publication dependent on the results; involvement of industry in study design/statistical analyses/manuscript preparation
Biological plausibility	Whether or not the SNP(s) have an identified mechanism of action relevant to the gene-diet association/interaction
Nutrient-dose response	Evidence that different doses of nutrients have different effects on the outcome of interest; nutrient-dose responses may be linear, j-shaped or u-shaped depending on the nutrient of interest
Allele-dose response	For single SNP studies, evidence that homozygotes exhibit a larger effect on the outcome of interest compared to heterozygotes. For polygenic studies, those with more risk/response alleles exhibit larger effects compared to those with fewer risk/response alleles.
Different levels of evidence identified	Based on the abovementioned factors, a conclusion is reached for the overall level or grade of evidence for a particular topic

## Results

### Systematic Review of Evidence Evaluation Frameworks

Upon screening 3,931 articles, a total of 49 articles were included in this systematic review (Figure 1) representing 41 unique frameworks (*n* = 19 used in genetics; *n* = 9 used in nutrition; *n* = 13 used in both genetics and nutrition). One framework that has been used for evidence evaluation in both nutrition and genetics has further been used for evidence evaluation in nutritional genomics (23). The main reasons for excluding studies were if an article did not include an overview of a specific framework that was intended for use in evaluating scientific validity of the body of evidence and not exclusively single studies/articles [e.g., (24–26)] or if the framework had not been used for scientific validity assessment in nutrition or genetics [e.g., (20, 27, 28)].

There was a wide variability in the factors considered when determining scientific validity in the existing frameworks (Tables 2, 3). Frameworks commonly considered study design and quality for evidence evaluation. Consideration of biological plausibility was more commonly considered in frameworks used in genetics than in nutrition. Effect size, statistical precision, and dose-response effects were rarely included as key components of evidence evaluation in both the fields of nutrition and genetics. For frameworks used in genetics, the scientific validity assessment was often part of a larger framework for evaluating genetic tests. For example, in addition to scientific validity assessment (a component of clinical validity), the ACCE framework provides guidance for evaluating analytical validity, clinical utility and ethical/legal/social implications (8). Typically, the evaluation of scientific validity was summarized into 3–4 categories indicating the overall level of evidence, such as “high,” “moderate,” or “low” quality, or “Grade A/B/C/D.”

**Table 2:** Summary of included evidence evaluation frameworks for determining scientific validity in nutrition and/or genetics.

**First Author, Year**	**Evidence grading framework title (Abbreviated)**	**Included study designs**	**Main scientific validity evaluation components described in the tool**	**Categories for levels of evidence, from highest to lowest**	**Brief description of system**	**Used in nutrition and/or genetics?**
Mechanick et al. (29)	AACE (original)	- RCTs - Multicenter trials - Meta-analyses - Prospective cohort study - Observational studies - Case series or case reports	- Study design and quality - Generalizability	Grade A Grade B Grade C Grade D	Methods used to determine levels of evidence (and practice recommendations) for developing AACE CPGs	Nutrition and genetics
Mechanick et al. (30)	AACE−2010 Update	- Meta-analyses - RCTs - Non-randomized trials - Prospective cohort study - Retrospective case-control study - Cross-sectional study - Surveillance study - Consecutive case series - Single case reports	- Study design and quality (premise correctness, allocation concealment, selection bias, appropriate blinding, using surrogate end points, sample size, null hypothesis vs. Bayesian statistics) - Data analysis (intent-to-treat, appropriate statistics) - Interpretation of Results (generalizability, logical, incompleteness, validity) - Consensus	Grade A Grade B Grade C Grade D	2010 update of the methods used to determine levels of evidence (and practice recommendations) for developing AACE CPGs	Nutrition and genetics
Mechanick et al. (31)	AACE−2014 Update	- Meta-analyses - RCTs - Non-randomized trials - Prospective cohort study - Retrospective case-control study - Cross-sectional study - Surveillance study - Consecutive case series - Single case reports	- Study design and quality (premise correctness, allocation concealment, selection bias, appropriate blinding, using surrogate end points, sample size, null hypothesis vs. Bayesian statistics) - Data analysis (intent-to-treat, appropriate statistics) - Interpretation of results (generalizability, logical, incompleteness, validity) - Consensus	Grade A Grade B Grade C Grade D	2017 update of the methods used to determine levels of evidence (and practice recommendations) for developing AACE CPGs	Nutrition and genetics
Mechanick et al. (32)	AACE−2017 Update	- Meta-analyses - RCTs - Non-randomized trials - Prospective cohort study - Retrospective case-control study - Nested case-control study - Cross-sectional study - Epidemiological study - Consecutive case series - Single case reports - Network meta-analyses - Nested case-control study - Open-label extension study - *Post-hoc* analysis study - Discovery science - Economic study - Preclinical study - Basic research	- Study design and quality (allocation concealment, blinding, comparator group, endpoints, hypothesis, power analysis, premise, Type I error) - Data analysis (Intent-to-treat, modeling, network analysis, statistics, appropriate follow-up, appropriate trial termination) - Interpretation of results (generalizability, incompleteness, logical, overstated, validity) - Consensus	Grade A Grade B Grade C Grade D	2017 update of the methods used to determine levels of evidence (and practice recommendations) for developing AACE CPGs	Nutrition and genetics
Centers for Disease Control and Prevention (CDC) (8)	ACCE	N/A (no quality assessment for study design)	- Clinical validity: sensitivity, specificity, prevalence, validation in target population, positive/negative predictive values, genotype/phenotype relationships, genetic/environmental, or other modifiers	N/A	A method used to evaluate genetic tests, which includes 44 questions relevant to the disorder/setting, analytical validity, clinical validity, clinical utility, and ethical/legal/social implications.	Genetics
Burke and Zimmern (33)	ACCE—Expanded	- Genetic association studies/primary research - Systematic reviews - Meta-analyses	- Clinical validity: assessment of link between genotype and disease, sensitivity, specificity, prevalence, validation in target population, positive/negative predictive values, genotype/phenotype relationships, genetic/environmental, or other modifiers	N/A	A method used to evaluate genetic tests, which includes questions relevant to the disorder/setting, analytical validity, clinical validity, clinical utility, and ethical/legal/social implications. Variation from the original ACCE Framework relevant to scientific validity includes an assessment of the link between genotype and disease such as through a systematic review of genetic association studies	Genetics
Calonge et al. (34)	ACHDNC	- Any studies included within systematic reviews	- Study design (strength) and quality - Consistency of results and interventions	Adequate Inadequate	A component of a larger framework used to evaluate conditions nominated for inclusion on newborn and/or childhood public health genetic screening panels	Genetics
Richards et al. (35)	ACMG/AMP	N/A (no quality assessment for study design)	- Computational evidence (using online databases, or *in silico* predictive programs) - Mechanism for pathogenicity/functional domain - Segregation - *De novo* inheritance - Family history - Prevalence in affected individuals vs. controls - Allele frequency	Pathogenic Likely pathogenic Uncertain significance Likely benign Benign With subcategories: Very strong Strong Moderate Supporting	Guidelines for the interpretation of sequence variants in genes that cause Mendelian disorders	Genetics
Owens et al. (36)	AHRQ	-Meta-analyses - RCTs - Systematic reviews - Observational studies - Diagnostic test studies	- Study design - Risk of bias - Consistency - Directness - Precision - Dose-response association - Confounders - Strength of association - Publication Bias	High Moderate Low Insufficient	An evidence evaluation method adapted from, and conceptually similar to, the GRADE approach	Nutrition
Strande et al. (37, 38)	ClinGen	- Gene-disease association studies (case-level, case-control and experimental)	**Genetic evidence:** Case-level data: - Variant's inheritance pattern - Molecular consequence - Evidence of pathogenicity in disease - Compelling segregation analysis Case-control data: - Variant detection methodology - Power - Bias and confounding factors Statistical power **Experimental evidence:** - Biochemical Function - Experimental protein interactions - Expression - Functional alteration - Phenotypic rescue - Model systems	- Definitive - Strong Moderate Limited No Reported Evidence Conflicting Evidence	A framework that uses a point system to classify gene-disease relationships by the quantity and quality of the evidence supporting such a relationship	Genetics
Merlin et al. (39)	Codependent Technologies Assessment	- Not stated	- Strength, specificity and temporality of association - Consistency and coherence of effect - Biological plausibility - Dose-response	N/A	A checklist for determining the clinical effectiveness of a codependent technology that includes consideration of context, clinical benefit, evidence translation, cost-effectiveness, and financial impact; used to determine national coverage or reimbursement decisions in Australia	Genetics
Caudle et al. (40)	CPIC	- All study designs including but not limited to: - RCTs with pharmacogenetic-based prescribing vs. standard dosing - Pre-clinical and clinical studies - Case studies - *In vivo* studies - *In vitro* studies	- Study design - Study conduct, power and quality - Number of studies - Consistency - Generalizability - Indirectness	High Moderate Weak	A method for summarizing pharmacogenomics evidence in order to tailor medication recommendations based on genetics.	Genetics
Ciesielski et al. (41)	DiCE	Omic, informatics, and experimental evidence	- Category of evidence (omic/observational, biological database/informatic or experimental) - Amount/validation (consistency) of evidence - Evidence of association between the factor and pathophysiology of the disease/condition - Statistical analysis	Strong (score of 6–10) Weak (score of <6)	A scoring system that can be used to determine if genetic research of complex diseases is strong or weak, based largely on study validation and evidence of biological plausibility.	Genetics
Treadwell et al. (42)	ECRI Group System	- Any studies included in systematic reviews/meta-analyses	- Study quantity - Study quality - Consistency - Robustness - Magnitude of Effect	Strength Rating: Strong Weak Moderate Inconclusive Stability Rating: High Stability Moderate Stability Low Stability Unstable	An evidence evaluation system that builds on existing systems by considering both quantitative and qualitative conclusions, strength and stability of evidence and a priori judgments	Nutrition
Teutsch et al. (43)	EGAPP	- Any peer-reviewed publication of original data or systematic review/meta-analysis of these studies - Peer-reviewed unpublished listerature (e.g., from FDA Advisory Committee Meetings) - Other sources on a case-by-case basis (e.g., unpublished data)	- Clinical validity (includes considering the disorder/phenotype and outcomes of interest, study design and test/methodology, study population, consistency, blind comparison, data analysis, publication bias, conflict of interest)	Convincing Adequate Inadequate	A method used to determine whether a genetic test should be used in practice, which includes consideration of the overarching question, analytic validity, clinical validity (the focus of the present review article), and clinical utility	Genetics
Veenstra et al. (44)	EGAPP—Update	- Any peer-reviewed publication of original data or systematic review/meta-analysis of these studies - Peer-reviewed unpublished listerature (e.g., from FDA Advisory Committee Meetings) - Other sources on a case-by-case basis (e.g., unpublished data)	- Clinical Validity (includes all aspects of the original EGAPP methods, but also assesses “fatal flaws” in studies and includes decision models during evidence review)	Convincing Adequate Inadequate	An updated EGAPP method aimed to improve efficiency and relevance, used to determine whether a genetic test should be used in practice, which includes consideration of the overarching question, analytic validity, clinical validity (the focus of the present review article), and clinical utility	Genetics
FDA (45)	FDA Guidelines for Scientific Evaluation of Health Claims	- Observational studies - Interventional studies - Review articles	- Number of studies and number of participants - Methodological quality - Outcome - Consistency - Relevance to population	Supports health claim Refutes health claim	The FDA's system for evaluating the body of scientific evidence specific to health claims	Nutrition
Hillier et al. (46)	FORM	- Systematic review of RCTs - RCTs - pseudo-RCT - Comparative studies with concurrent controls - Cohort studies - Case-control studies - Interrupted time series studies with or without a control group - Single arm studies - Case series	- Evidence base - Consistency - Clinical impact - Generalizability - Applicability	Grade A (Excellent) Grade B (Good) Grade C (Satisfactory) Grade D (Poor)	A framework for use by clinical practice guideline developers to determine the strength of recommendation based on the body of evidence	Nutrition and genetics
Rousseau et al. (47)	GETT	- N/A (no quality assessment for study design)	- Clinical validity: diagnostic specificity, sensitivity, positive, and negative predictive values in target population	N/A	A 72-item checklist to be used when determining if a genetic test should be implemented in a practice setting	Genetics
Guyatt et al. (48, 49)	GRADE	- Any observational or interventional study	- Study design - Risk of bias - Inconsistency of results - Indirectness of evidence - Imprecision - Publication bias - Magnitude of effect - Dose-response gradient - Confounders likely to minimize the effect	High (A/⊕⊕⊕⊕) Moderate (B/⊕⊕⊕○) Low (C/⊕⊕○○) Very Low (D/⊕○○○)	A process for rating the quality of scientific evidence and developing recommendations for healthcare.	Nutrition and genetics
Lewin et al. (50) Munthe-Kaas et al. (51) Colvin et al. (52)	GRADE-CERQual	- Systematic reviews of qualitative studies	- Methodological limitations - Coherence - Adequacy of data - Relevance	High confidence Moderate confidence Low confidence Very low confidence	Series of articles describing the GRADE-CERQual tool for use in evaluating confidence in the evidence from systematic reviews of qualitative evidence	Nutrition
Ioannidis et al. (53)	HuGENet	- Epidemiological evidence of genetic associations	- Study design - Study quality - Amount of evidence - Replication - Protection from bias	Strong evidence Moderate evidence Weak evidence	A proposed framework from the HuGENet working group for assessing the cumulative evidence for genetic associations	Genetics
Callahan et al. (54)	HyQue	- N/A	- Domain specific rules (triggered by types of events described in the hypothesis input) - System rules (triggered by output based on domain-specific rules and operators that link events in the hypothesis)	Score between 0 and 1 (higher evaluation/hypothesis scores are indicative of greater evidence)	Algorithm-based tool used to generate experimental and biological hypotheses related to the role of genes in aging-related biological processes using Semantic Web; a rule-based system used to obtain and evaluate evidence using various technologies	Genetics
WHO (55)	IARC	- Experimental and observational studies in humans and animals - Mechanistic studies	- Study design and quality - Risk of bias - Confounding - Effect sizes - Consistency - Biological plausibility	- Sufficient evidence of carcinogenicity - Limited evidence of carcinogenicity - Inadequate evidence of carcinogenicity - Evidence suggesting lack of carcinogenicity (stratified into *Carcinogenicity in Experimental Animals* or *Carcinogenicity in Humans*)	A method used to develop monographs for evidence of the carcinogenicity of various agents including lifestyle factors	Nutrition and genetics
Greer et al. (56)	ICSI Guidelines	- RCTs - Cohort studies - Case-control studies - Nonrandomized trial - Cross-sectional study - Meta-analyses - Narrative review - Uncontrolled case series	- Design type - Study quality - Class of research report - Population studied/Sample size - Primary outcomes Measure(s)/Results - Authors' conclusions	Grade I: Good evidence Grade II: Fair evidence Grade III: Limited evidence Grade IV: Opinion	An approach to evidence grading described by the working group as “practical,” which can be used to evaluate evidence relevant to healthcare professionals	Nutrition
Boffetta et al. (57)	No Title	- Systematic reviews/meta-analyses - Any original research of gene x environment interactions	- Evidence of main effects of a) environmental exposure and b) genetic variant on outcome of interest, as well as evidence of interaction between exposure and genetic variant (includes consideration of study quality, consistency, power, confounding, bias, dose-response, biological plausibility, effect size, measurement error, and imprecision) - Amount of evidence - Replication - Protection from bias	Strong Moderate Weak	Guidelines for evaluating the body of evidence related to gene-environment interactions relevant to human carcinoma; the framework can also be applied to other chronic diseases.	Nutrition and genetics
Burke et al. (58)	No Title	- Not stated	- Evidence of causal association - Prevalence - Positive and negative predictive value	N/A	A framework used for evaluating the use of genetic testing to screen for adult-onset chronic diseases	Genetics
McShane et al. (59)	No Title		- Data quality - Study design and quality - Data processing and statistical methods - Validation - Applicability to patient population	N/A	A checklist for evaluating the cumulative evidence for using an omic predictor to guide patient therapy; includes multiple components beyond scientific validity assessment.	Genetics
Senol-Cosar et al. (60)	No Title	- Genetic association studies including case-control - Meta-analyses	- Study design/data quality - Associated phenotypes - Replication - Mechanism of action/function	Established risk allele Likely risk allele Uncertain risk allele	A framework for determining the validity of evidence for risk alleles in disease, based on the ACMG/AMP framework. This framework is intended to be used to decide one return of results in a clinical or research setting	Genetics
Schwingshackl et al. (61)	NutriGrade	Meta-Analyses	- Risk of bias, study quality and study limitations - Precision - Heterogeneity - Directness - Publication bias - Funding bias - Study design - Effect size - Dose response	High (score of 8–10) Moderate (score of 6–7.99) Low (score of 4–5.99) Very Low (score of 0–3.99)	A 10-point scoring system used to evaluate the quality of evidence for meta-analyses of RCTs or cohort studies.	Nutrition
University of Oxford (62)	OCEBM	-Systematic reviews - Randomized trials - Non-randomized trials - Cohort - Case series - Case-control - Cross sectional - Mechanistic reasoning studies	- Prevalence - Accuracy of diagnostic tests - Prognosis - Treatment benefits - Treatment harms - Usefulness of screening	Level 1 Level 2 Level 3 Level 4 Level 5	A method of evaluating the evidence, aimed to assist clinicians, researchers, and/or patients to find the likely best evidence when choosing a treatment	Nutrition and genetics
Practice-Based Evidence in Nutrition (PEN) (12)	PEN Evidence Grading Checklist	- Meta-analyses - Systematic reviews - RCTs - Non-randomized trials - Trial that used historical controls - Case-control studies - Cohort studies - Cross-sectional studies - Case reports or case series - Ecological studies	- Evidence - Consistency - Clinical impact - Generalizability - Applicability	Grade A Grade B Grade C Grade D	An evidence evaluation tool designed for assessing and summarizing nutrition research in order to inform nutrition practice recommendations.	Nutrition
Whirl-Carrillo et al. (63, 64)	PharmGKB	- Meta-analyses - Clinical studies - GWAS - Cohort studies - Case report - *in vitro*/molecular/functional assay studies	- FDA-approved drug label annotation - Functional SNPs/biological plausibility - *P*-value - Cohort size - Effect size - Study type - Association and significance	Level 1A (High) Level 1B (High) Level 2A (Moderate) Level 2B (Moderate) Level 3 (Low) Level 4 (Unsupported)	A scoring system used to determine the level of evidence for pharmacogenomic research	Genetics
Whirl-Carillo et al. (65)	PharmGKB – 2021 Update	- Meta-analyses - Clinical studies - GWAS - Cohort studies - Case report - *in vitro*/molecular/functional assay studies	- FDA-approved drug label annotation - Phenotype category - *P*-value - Cohort size - Effect size - Study type - Association and significance	Level 1A (High) Level 1B (High) Level 2A (Moderate) Level 2B (Moderate) Level 3 (Low) Level 4 (Unsupported)	An updated quantitative scoring system used to determine the level of evidence for pharmacogenomic research	Genetics
Harbour et al. (66)	SIGN	- Systematic reviews and meta-analyses - RCTs - Cohort - Case-control - Case reports - Case series - Diagnostic - Economic - Expert opinion	- Study design - Study quality - Applicability to target patient group - Consistency	1++ 1+ 1– 2++ 2+ 2– 3 4	A component of SIGN's more broad methods for developing guidelines for clinical practice that is focused on determining levels of evidence primarily based on study design and study quality.	Nutrition and genetics
Ebell et al. (67)	SORT	- Systematic reviews and meta-analyses -RCTs - Clinical trials - Cohort studies - Case-control studies - Case series	- Study quality - Type of outcomes - Number, consistency and coherence of Evidence - Benefits, harms, and costs	Grade A (Based on consistent and good-quality patient-oriented evidence) Grade B (Based on inconsistent or limited-quality patient-oriented evidence) Grade C (Based on consistent and good-quality patient-oriented evidence)	A patient-centered approach (i.e., focused on evidence measuring outcomes that matter to patients) for rating the strength of healthcare recommendations that considers quality, quantity and consistency of evidence	Nutrition and genetics
Hornberger et al. (68)	SynFRAME	- Primary research including controlled trials	- Study design - Sample population - Clinical meaningfulness - Statistical significance	N/A	A comprehensive framework for evaluating laboratory-developed tests, which includes consideration of analytic validity, clinical validity, clinical utility, economic, and social implications and presentation	Genetics
Harris et al. (69)	USPSTF Method	- Systematic reviews - Case-control studies - RCTs - Cohort studies - Diagnostic accuracy studies	- Individual study quality - Linkage in the analytic framework - Entire preventive service	Good Fair Poor	Evidence grades are used to determine if a particular component of health care (e.g., a disease-risk genetic test) should be provided in practice or not.	Nutrition and genetics
Guirguis-Blake et al. (70, 71)	USPSTF−2007 Update	- Any omics study	- Study design - Internal validity - External validity - Precision - Consistency of results - Additional factors (e.g., dose response, biological plausibility)	High Moderate Low	Evidence grades are used to determine if a particular component of health care (e.g., a disease-risk genetic test) should be provided in practice or not.	Nutrition and genetics
World Cancer Research Fund (72)	WCRF	- RCTs - Non-randomized controlled trials - Observational studies - Epidemiological studies - Case-control studies - Cross-sectional studies - Clinical and laboratory investigations	- Study design - Study quality - Consistency - Dose-response - Biological plausibility	Strong (convincing, probable, or substantial effect on risk unlikely) Limited (limited—suggestive, limited—no conclusion)	A component of a larger guideline for determining evidence-based policy and practice globally related to lifestyle (nutrition and physical activity) and cancer associations.	Nutrition
World Health Organization (73)	WHO Methods	- RCTs - Case-control studies - Cohort studies - Descriptive studies - Migrant studies - Ecological studies - Case reports	- Study design - Study quality - Consistency - Biological plausibility	Convincing evidence Probable evidence Possible evidence Insufficient evidence	A component of a larger report on nutrition and chronic disease prevention; the methods for determining scientific validity are based off the WCRF methods.	Nutrition

*AACE, American Association of Clinical Endocrinologists; ACCE, Analytic and Clinical validity, Clinical utility and associated Ethical; ACHDNC, Advisory Committee on Heritable Disorders in Newborns and Children; ACMG/AMP, American College of Medical Genetics and Genomics/Association for Molecular Pathology; AHRQ, Agency for Healthcare Research and Quality; CAT, Companion Test Assessment Tool; CDC, Center for Disease Control and Prevention; ClinGen, Clinical Genome Resource; CPG, Clinical Practice Guidelines; CPIC, Clinical Pharmacogenetics Implementation Consortium; DiCE, Diverse Convergent Evidence Score; ECRI, Emergency Care Research Institute; EGAPP, The Evaluation of Genomic Applications in Practice and Prevention; FDA, Food and Drug Administration; FORM: abbreviation not specified in article; G × E: gene-environment; GETT, Genetic testing Evidence Tracking Tool; GRADE, The Grading of Recommendations Assessment, Development and Evaluation; GRADE-CERQual, The Grading of Recommendations Assessment, Development and Evaluation-Confidence in the Evidence from Reviews of Qualitative research; GWAS, genome-wide association study; HuGENet, The Human Genome Epidemiology Network; HyQue, Semantic Web tool for hypothesis-based querying and evaluation; IARC, International Agency for Research on Cancer; ICSI, Institute for Clinical Systems Improvement; N/A, not applicable; SORT, Strength of Recommendation Taxonomy; OCEBM, Oxford Center for Evidence-Based Medecine; PEN, Practice-Based Evidence in Nutrition; PharmGKB, The Pharmacogenomics Knowledge Base; SIGN, Scottish Intercollegiate Guidelines Network; SNP, single nucleotide polymorphism; SORT, Strength of recommendation taxonomy; SynFRAME, Synthesized Frameworks; USPSTF, United States Preventive Services Taskforce; WHO, World Health Organization; WCRF, World Cancer Research Fund*.

**Table 3:** Categorization matrix to determine the appropriateness of existing tools for evaluating the scientific validity of nutrigenetic interactions.

**Factors considered for evaluating the body of evidence →** **Abbreviated name of framework ↓**	**QA for study ROB/Methodological quality**	**Different study designs included (with different weighting by design)**	**Population directness/Generalizability**	**Study directness (Relatedness)**	**Statistical precision**	**Consistency of study results**	**Plausible confounding**	**Effect size**	**Publication/Funding bias**	**Biological plausibility**	**Nutrient-dose response**	**Allele-dose response**	**Different levels of evidence identified**
AACE (original) (29)	**✓**	3	**✓**	**✓**	**X**	**✓**	**–**	**X**	**X**	**X**	**X**	**X**	**✓**
AACE−2010 Update (30)	**✓**	3	**✓**	**✓**	**X**	**✓**	**–**	**X**	**X**	**X**	**X**	**X**	**✓**
AACE−2014 Update (31)	**✓**	3	**✓**	**✓**	**–**	**✓**	**–**	**–**	**X**	**X**	**X**	**X**	**✓**
AACE−2017 Update (32)	**✓**	3	**✓**	**✓**	**–**	**✓**	**–**	**–**	**X**	**X**	**X**	**X**	**✓**
ACCE (8)	**X**	X	**✓**	**X**	**X**	**X**	**X**	**X**	**X**	**X**	**X**	**X**	**X**
ACCE—Expanded (33)	**X**	X	**✓**	**X**	**X**	**X**	**X**	**X**	**X**	**X**	**X**	**X**	**X**
ACHDNC (34)	**✓**	3	**X**	**✓**	**✓**	**✓**	**✓**	**X**	**X**	**X**	**X**	**X**	**✓**
ACMG/AMP (35)	**X**	X	**X**	**X**	**✓**	**X**	**✓**	**✓**	**X**	**✓**	**X**	**X**	**✓**
AHRQ (36)	**✓**	3	**X**	**✓**	**✓**	**✓**	**✓**	**✓**	**✓**	**X**	**✓**	**X**	**✓**
Boffetta et al. (57)	**✓**	3	**✓**	**✓**	**✓**	**✓**	**✓**	**✓**	**✓**	**✓**	**✓**	**X**	**✓**
Burke et al. (58)	**X**	X	**X**	**X**	**X**	**X**	**X**	**X**	**X**	**X**	**X**	**X**	**X**
ClinGen (37, 38)	**✓**	3	**✓**	**✓**	**X**	**✓**	**✓**	**✓**	**X**	**✓**	**X**	**X**	**✓**
Codependent technologies assessment (39)	**✓**	X	**X**	**X**	**X**	**✓**	**X**	**X**	**X**	**✓**	**X**	**X**	**X**
CPIC (40)	**✓**	3	**✓**	**✓**	**X**	**✓**	**✓**	**✓**	**X**	**✓**	**X**	**X**	**✓**
DiCE (41)	**X**	3	**X**	**–**	**–**	**✓**	**✓**	**X**	**X**	**✓**	**X**	**X**	**✓**
ECRI group system (42)	**✓**	3	**X**	**✓**	**✓**	**✓**	**X**	**✓**	**✓**	**X**	**X**	**X**	**✓**
EGAPP (43)	**✓**	3	**✓**	**✓**	**X**	**✓**	**X**	**✓**	**✓**	**X**	**X**	**X**	**✓**
EGAPP update (44)	**✓**	3	**✓**	**✓**	**X**	**✓**	**X**	**✓**	**✓**	**X**	**X**	**X**	**✓**
FDA guidelines for scientific evaluation of health claims (45)	**✓**	3	**✓**	**X**	**X**	**✓**	**✓**	**✓**	**X**	**✓**	**X**	**X**	**✓**
FORM (46)	**✓**	3	**✓**	**✓**	**✓**	**✓**	**–**	**✓**	**X**	**X**	**X**	**X**	**✓**
GETT (47)	**X**	X	**✓**	**X**	**X**	**X**	**X**	**X**	**X**	**X**	**X**	**X**	**X**
GRADE (48, 49)	**✓**	3	**✓**	**✓**	**✓**	**✓**	**✓**	**✓**	**✓**	**X**	**✓**	**X**	**✓**
GRADE-CERQual (50–52)	**✓**	X	**X**	**X**	**X**	**✓**	**X**	**X**	**X**	**X**	**X**	**X**	**✓**
HuGENet (53)	**✓**	3	**✓**	**✓**	**X**	**✓**	**✓**	**✓**	**X**	**X**	**X**	**X**	**✓**
HyQue (54)	**X**	–	**X**	**X**	**X**	**✓**	**X**	**–**	**X**	**✓**	**X**	**X**	**✓**
IARC (55)	**✓**	3	**X**	**✓**	**✓**	**✓**	**✓**	**✓**	**X**	**✓**	**✓**	**X**	**✓**
ICSI guidelines (56)	**✓**	3	**✓**	**X**	**✓**	**✓**	**X**	**✓**	**X**	**X**	**X**	**X**	**✓**
McShane et al. (59)	**✓**	3	**✓**	**✓**	**X**	**✓**	**✓**	**–**	**X**	**X**	**X**	**X**	**X**
NutriGrade (61)	**✓**	X	**✓**	**✓**	**✓**	**✓**	**✓**	**✓**	**✓**	**X**	**✓**	**X**	**✓**
OCEBM (62)	**✓**	3	**✓**	**✓**	**✓**	**✓**	**✓**	**✓**	**X**	**✓**	**X**	**X**	**✓**
PEN evidence grading checklist (12)	**✓**	3	**✓**	**X**	**X**	**✓**	**✓**	**✓**	**X**	**X**	**X**	**X**	**✓**
PharmGKB (63, 64)	**X**	3	**X**	**–**	**✓**	**✓**	**X**	**✓**	**X**	**✓**	**X**	**X**	**✓**
PharmGKB−2021 Update (65)	**X**	3	**X**	**–**	**✓**	**✓**	**X**	**✓**	**X**	**✓**	**X**	**X**	**✓**
Senol-Cosar et al. (60)	**✓**	3	**X**	**X**	**✓**	**✓**	**X**	**✓**	**X**	**✓**	**X**	**X**	**✓**
SIGN (66)	**✓**	3	**✓**	**X**	**✓**	**✓**	**✓**	**✓**	**–^a^**	**X**	**X**	**X**	**✓**
SORT (67)	**✓**	3	**✓**	**X**	**X**	**✓**	**X**	**X**	**X**	**X**	**X**	**X**	**✓**
SynFRAME (68)	**✓**	3	**✓**	**✓**	**X**	**✓**	**✓**	**X**	**X**	**X**	**X**	**X**	**X**
USPSTF Method (69)	**✓**	3	**✓**	**✓**	**X**	**✓**	**✓**	**✓**	**X**	**✓**	**X**	**X**	**✓**
USPSTF−2007 Update (70, 71)	**✓**	3	**✓**	**✓**	**✓**	**✓**	**✓**	**✓**	**X**	**✓**	**X**	**X**	**✓**
WCRF (72)	**✓**	3	**✓**	**✓**	**✓**	**✓**	**✓**	**✓**	**✓**	**✓**	**✓**	**X**	**✓**
WHO methods (73)	**✓**	3	**X**	**X**	**X**	**✓**	**✓**	**✓**	**X**	**✓**	**X**	**X**	**✓**

***✓**, yes (included in framework); X, no (not included in framework); –, cannot determine*.

*AACE, American Association of Clinical Endocrinologists; ACCE, Analytic and Clinical validity, Clinical utility and associated Ethical; ACHDNC, Advisory Committee on Heritable Disorders in Newborns and Children; ACMG/AMP, American College of Medical Genetics and Genomics/Association for Molecular Pathology; AHRQ, Agency for Healthcare Research and Quality; CAT, Companion Test Assessment Tool; CDC, Center for Disease Control and Prevention; ClinGen, Clinical Genome Resource; CPG, Clinical Practice Guidelines; CPIC, Clinical Pharmacogenetics Implementation Consortium; DiCE, Diverse Convergent Evidence Score; ECRI, Emergency Care Research Institute; EGAPP, The Evaluation of Genomic Applications in Practice and Prevention; FDA, Food and Drug Administration; FORM, abbreviation not specified in article; G × E, gene-environment; GETT, Genetic testing Evidence Tracking Tool; GRADE, The Grading of Recommendations Assessment, Development and Evaluation; GRADE-CERQual, The Grading of Recommendations Assessment, Development and Evaluation-Confidence in the Evidence from Reviews of Qualitative research; GWAS, genome-wide association study; HuGENet, The Human Genome Epidemiology Network; HyQue, Semantic Web tool for hypothesis-based querying and evaluation; IARC, International Agency for Research on Cancer; ICSI, Institute for Clinical Systems Improvement; SORT, Strength of Recommendation Taxonomy; OCEBM, Oxford Center for Evidence-Based Medecine; PEN, Practice-Based Evidence in Nutrition; PharmGKB, The Pharmacogenomics Knowledge Base; QA, quality assessment; ROB, risk of bias; SIGN, Scottish Intercollegiate Guidelines Network; SNP, single nucleotide polymorphism; SORT, Strength of recommendation taxonomy; SynFRAME, Synthesized Frameworks; USPSTF, United States Preventive Services Taskforce; WHO, World Health Organization; WCRF, World Cancer Research Fund*.

a *Funding bias is included in the RCT methodology checklist, but it is not clear whether or how funding bias should be considered in the overall evidence evaluation*.

### Categorization Matrix Development

Important factors (matrix headings) identified by the expert working group and external expert panel for evaluating nutrigenetic evidence related to: study design and quality, generalizability, directness, consistency, precision, confounding, effect size, biological plausibility, publication/funding bias, allele and nutrient dose-response, as well as summary levels of evidence. The categorization matrix is detailed in Table 3 and a description of each key factor (column heading) is described in Table 1.

It should be noted that only brief descriptions are provided in Table 1 and many of the factors incorporate consideration of several sub-factors. For example, in a risk of bias assessment of individual studies, the quality of data collection methods, statistical analyses, and sample size would be taken into consideration, among other factors (74). The factors were identified through expert consultation as well as the literature search. For example, it has been suggested in the literature that there is stronger evidence of clinical validity when a gene-disease relationship is biologically plausible, or a mechanism of action is well-understood (14, 37, 75). Similar views have been highlighted in the field of nutrigenetics (20) and medicine (76). Unknown confounding factors can result in erroneous correlations, but if a causal mechanism can provide a plausible explanation for the correlation, we increase our confidence that the correlation could be causal (76). Moreover, evidence of biological plausibility can lead to improved generalizability (77); if a function is altered, ethnicity is less likely to influence this function. However, epistasis is plausible (78), and another SNP that may be more prevalent in certain ethnicities could alter the effects of a functional SNP; epistasis is a factor to consider when evaluating the broader topic of biological plausibility. For example, a 4-SNP haplotype in leukotriene A4 hydrolase can modify the risk of myocardial infarction dependent on ethnicity (79). This factor was then presented to the expert group for feedback, and was well-received. Therefore, evidence of biological plausibility (functional SNPs) and epistasis were included in the categorization matrix. Ethnicity was also included in the matrix, falling within the category titled “Population Directness/Generalizability.” Related, evidence of allele-dose response was also included in the matrix given that it is expected for homozygotes to have an enhanced response compared to heterozygotes for a specific allele, or in the case of polygenic studies, an increasing number of risk/response alleles would result in a greater risk/response [e.g., (80–82)]. Some critiques of existing evidence evaluation frameworks were also considered when developing the categorization matrix including: limited criteria on validity (including concurrent and external validity), reliability, inadequacy addressing non-RCTs, as well as balancing simplicity and comprehensiveness/clarity (19, 83).

### Appropriateness of Existing Frameworks for Nutrigenetic Evidence Evaluation

The categorization matrix was used to determine which, if any, of the existing might be most appropriate for comprehensive nutrigenetic evidence evaluation. Significantly, this systematic review did not identify any frameworks that considered all the factors deemed important by the expert group in evidence evaluation for nutritional genomics. However, there were two identified tools that considered all but one key factor relevant to evidence evaluation in nutritional genomics—the methods used by the World Cancer Research Fund (WCRF) and the gene-environment interaction framework developed by Boffetta et al. (57, 72). The factor missing from both of these methods was consideration of evidence of allele-dose responses when determining levels of evidence (57, 72). It is interesting to note that both frameworks were developed specifically for use in evidence evaluation related to cancer outcomes (57, 72). Boffetta et al.'s framework is similar to the WCRF, and the WCRF authors suggest that WCRF systematic reviews should be used (when possible) to score the strength of the evidence using this framework (57). The framework of Boffetta et al. was unique from others identified in this review as it was specific to gene-environment interactions, including gene-diet interactions. Additionally, while it was developed specifically for cancer outcomes, the authors note that it can also be applied to different health outcomes (57). Given its direct relevance to nutrigenetics, its previous use in this field (23), and applicability to broader health outcomes, this framework (57) was deemed more appropriate for nutrigenetic evidence evaluation compared to the WCRF methods (72).

We propose the use of Boffetta et al.'s framework (57), with some minor modifications, for use in broad (i.e., beyond cancer outcomes) nutrigenetics evidence evaluation. The use of this framework would result in the classification of nutrigenetic evidence into three categories: strong, moderate, or weak.

### Proposed Modifications to Boffetta et al.'s Framework

Four minor modifications to this framework are suggested. First, (i) evidence of allele-dose responses should increase our confidence in a gene-diet interaction effect. This could be integrated into Step 4 of the framework as it is considered an “additional support for [the] interaction” (57). Additionally (ii), it would be beneficial to provide guidance on how individual studies can be used for evidence evaluation when systematic reviews do not exist. Further (iii), it should also be evident that this framework can be used for evaluation of topics beyond cancer occurrence, and how the guidelines may change when evaluating evidence for other outcomes (e.g., identifying resources beyond the WRCF systematic reviews and IARC monographs). Last (iv), it could be beneficial to provide a title for this framework for ease of reference. As the authors suggest, these guidelines are considered interim recommendations and their performance should be further tested in outcomes beyond cancer occurrence (57).

## Discussion

This review provides an overview of one key aspect to consider when evaluating the suitability of nutrigenetic tests for clinical practice—scientific validity. While we propose that Boffetta et al. (57) have developed the most appropriate framework for determining the scientific validity of gene-diet associations and interactions, an important next step is to consider how we should then use summaries of scientific validity to develop clinical best practice guidelines. Further components of genetic testing evaluation may include clinical utility, analytic validity, ethical/legal/social implications, and others (18). In nutrition, dietary interventions may also be evaluated in the context of patient views/preferences, facilitators and barriers to their application, resource implications, and others (13). Future research should seek to explore these additional evaluation components and determine how nutrigenetic interventions should be evaluated within these contexts. It should be noted that while genetic variability is one key aspect of precision nutrition, multiple other environmental, and personal factors can influence health and disease risk (84). Examples include physical activity levels, the gut microbiome, and the metabolome (84). As such, systems biology framework is necessary for both nutrigenetic research and when developing clinical best practice guidelines in precision nutrition. Furthermore, if a genetic test lacks clinical (including scientific) validity, there is typically little need to proceed with assessing other aspects of utility/validity as there is insufficient evidence to support the particular test for clinical use (44). In addition, a care map, which outlines step-by-step directions for the appropriate integration of nutrigenetic testing into clinical practice, has been developed (3). This provides a broad overview of factors to consider to promote ethical and evidence-based practice in the field (3).

The framework developed by Boffetta et al. has been used to evaluate the evidence for nutritional genomics and breast cancer, lung cancer, prostate cancer, colorectal cancer, and stomach cancer outcomes (23). This previously conducted review identified one gene-diet interaction with moderately strong evidence (Grade BBB)—the interaction between the 10p14 locus and processed meat consumption on colorectal cancer risk (23, 85). CPG developers should now use these results to determine the possible suitability (or not) for testing this gene-diet interaction in clinical practice setting, using established CPG methods (7, 13). Additionally, the Boffetta et al. framework should now be used to determine levels of evidence for nutrigenetic outcomes beyond those related to cancer, while incorporating the modified methods described herein. This framework could be used on nutrigenetic topics deemed priorities, perhaps using a similar strategy for topic selection as that employed by the Evaluation of Genomic Application in Practice and Prevention (EGAPP) group, further discussed below (44). Some example topics for consideration may include the impact of caffeine on anxiety or other psychological conditions, based on genetic variation (86–89), or the impact of *FTO* genetic variation on weight and body composition responses to dietary protein intake (90, 91).

Researchers have highlighted the need for evidence syntheses and critical appraisal of evidence for years, in order to guide evidence-based nutrigenetic testing (92). Despite this, systematic reviews with evidence grading of gene-diet associations are limited, and there are varying opinions about the scientific validity of certain available nutrigenetic tests (5, 6). It is important to note that searches conducted for identifying articles relevant to a particular topic must be systematic and comprehensive in order to avoid any biases in the included studies for a particular review. It is worth highlighting that current practice guidelines for *MTHFR* genetic testing were not based on a systematic search, qualitative synthesis or evidence grading method (93); this could lead to biases in the guidelines. Given the extensive list of possible topics for evidence synthesis and evaluation, prioritization of nutrigenetic topics is needed. The EGAPP group prioritizes systematic review topics based on topic nomination by EGAPP members, stakeholder groups, steering committees, external consultants, Office of Public Health Genomics staff, and outside stakeholders (44). EGAPP then uses the results of these systematic reviews to make practice recommendations (either “recommendation for,” “recommendation against,” or “insufficient evidence” to make a recommendation) (44). A similar approach could work for evidence evaluation and clinical practice recommendations in nutritional genomics.

Overall, this review provided several novel insights for implementation science in nutrition, genetics, and nutrigenetics. Frameworks were variable in their consideration of biological plausibility, with more genetic frameworks considering this factor compared to nutrition frameworks. In the field of nutrigenetics, biological plausibility was deemed a key factor that should be integrated in evidence evaluation during the development of the categorization matrix. In genetics, the term “functional SNP” is used to describe SNPs with known physiological mechanisms of action (or those with a biologically plausible gene-environment function) (20, 94). The Clinical Genome Resource (ClinGen) Framework suggests that evidence of a functional variant contributes to an increasing strength of evidence (37). More specifically the strength of evidence increases depending on the evidence type. Evidence of a biochemical function, protein interaction, or expression is considered the lowest level of evidence, followed by evidence from cells of affected individuals or engineered cells as a higher level of evidence, and animal models, cell culture model systems, rescue in animal model, or rescue in engineered equivalent as the highest level of evidence (37). In this framework, evidence of function is scored, with a maximum possible score of six (37). Other frameworks and commentaries have also suggested that biological plausibility increases confidence in the evidence (14, 37, 75). Such evidence can additionally enhance the generalizability of research results; if a function is altered, ethnicity should not influence this function. However, it should also be noted that epistasis is plausible (78), and another SNP that may be more prevalent in certain ethnicities could alter the effects of a functional SNP. Overall, while biological plausibility was often not considered in existing frameworks, it was deemed important for nutrigenetic evidence evaluation.

There are some limitations and strengths of the present review. While we systematically searched the evidence, we cannot preclude the possibility that some evidence evaluation frameworks were missed. This is a possible limitation. In addition, while our methods were sound for developing the categories within the matrix, this is only a first iteration, and it is possible that future iterations may include different categories. For example, researchers have pointed out that genetic architecture matters in terms of the effect size of SNPs; ancestry-informative markers can provide a more detailed characterization of populations (79). Thus, consideration of how SNPs act within a particular genetic architecture may also be important to include in nutrigenetic evidence evaluation. Moreover, our search was limited to frameworks that have been previously used to evaluate evidence in nutrition and/or genetics, but other frameworks specific to genetics and nutrigenetics have been developed (20, 95). Future research should now seek to use these frameworks in order to pilot test their use for evidence evaluation in genetics and nutrigenetics and troubleshoot any issues. In addition, to our knowledge two frameworks exist that have been specifically developed for evaluating levels of evidence for gene-diet associations and interactions (20, 57). Only one of these frameworks met the inclusion criteria for the present review (57). The framework from Grimaldi et al. (20) was excluded given that it has not been used in a subsequent systematic review of nutrigenetic interactions for evidence evaluation thus far. This could be due to limitations of its use. Furthermore, the categorization matrix was developed while prioritizing comprehensiveness over simplicity, to determine the most thorough method for nutrigenetic evidence evaluation. This is considered a strength of the present work. More complex systems tend to be viewed as more rigorous. For example, evidence evaluation assessors have perceived GRADE as a more complex but highly rigorous system (96). It should be noted that frameworks which did not incorporate the key criteria deemed important by the expert panel, may still be highly rigorous for use in other fields, beyond nutritional genomics.

Overall, we have provided a synthesis of 49 articles representing 41 total frameworks that have been used to evaluate scientific validity in nutrition and genetics. Based on our evaluation of existing frameworks, it appears that Boffetta et al. have developed the most suitable and rigorous framework for use in evaluating the scientific validity of various gene-diet associations and interactions (57). Some minor modifications may help strengthen this framework further.

## Data Availability Statement

The original contributions presented in the study are included in the article/Supplementary Material, further inquiries can be directed to the corresponding author/s.

## Author Contributions

M-CV, RG, and JK conceptualized the review and developed the methods. M-CV and RG provided supervision for the project. DZ-M took primary responsibility on developing the search strategy, with guidance from JK. JK and VG were responsible for article screening and selection, as well as summarizing included articles and completing the categorization matrix, with assistance from EG and JM-B. JK wrote the first article draft. RH, DZ-M, VG, M-CV, RG, and JK revised the manuscript. All authors approved the final manuscript.

## Funding

JK received postdoctoral fellowships from the Canadian Institutes of Health Research, INAF, and NUTRISS. M-CV holds a Tier 1 Canada Research Chair in Nutrition Applied to Genetics and Metabolic Health.

## Conflict of Interest

RH was employed by company Human Longevity, Inc. RG has received compensation for advising the following companies: AIA, Embryome, Genomic Life, Grail, Humanity, Kneed Media, OptumLabs, Plumcare, Verily; and is co-founder of Genome Medical, Inc, a technology and services company providing genetics expertise to patients, providers, employers and care systems. The remaining authors declare that the research was conducted in the absence of any commercial or financial relationships that could be construed as a potential conflict of interest.

## Publisher's Note

All claims expressed in this article are solely those of the authors and do not necessarily represent those of their affiliated organizations, or those of the publisher, the editors and the reviewers. Any product that may be evaluated in this article, or claim that may be made by its manufacturer, is not guaranteed or endorsed by the publisher.
