# Supplementary material for: A Systematic Review and Recommendations Around Frameworks for Evaluating Scientific Validity in Nutritional Genomics
Source: Front Nutr. 2021 Dec 14;8:789215. doi: 10.3389/fnut.2021.789215 (PMC8728558; doi:10.3389/fnut.2021.789215)
Supplement: Supplementary file 1 [file Table_1.docx]

**Supplementary Table 1. Comprehensive search terms and search strategy**

Search Engine: Web of Science

| **Query** |
| --- |
| **TOPIC:** (("Methodology checklist" or "scoring system" or "grading method*" or "scientific validity" or "clinical validity" or "appraisal tool" or "critical appraisal" or "grading quality" or assessment or "quality indicator*" or evaluation or evaluating or "methodological quality" or grading or strength or framework) near/5 ("clinical studies" or "preclinical studies" or evidence or "medical studies") ) *AND* **TOPIC:** (("evidence based method" or "evidence based standard" or "evidence based medicine") )  **Refined by:** **DOCUMENT TYPES:** ( ARTICLE OR BOOK CHAPTER OR PROCEEDINGS PAPER ) AND **LANGUAGES:** ( ENGLISH OR FRENCH ) AND [excluding] **WEB OF SCIENCE CATEGORIES:** ( COMPUTER SCIENCE SOFTWARE ENGINEERING OR ENVIRONMENTAL SCIENCES OR EDUCATION SCIENTIFIC DISCIPLINES OR COMPUTER SCIENCE ARTIFICIAL INTELLIGENCE OR LAW OR LINGUISTICS OR HISTORY PHILOSOPHY OF SCIENCE OR MATHEMATICAL COMPUTATIONAL BIOLOGY OR SOCIAL WORK OR ACOUSTICS OR AGRICULTURE DAIRY ANIMAL SCIENCE OR BIOLOGY OR COMPUTER SCIENCE CYBERNETICS OR ECOLOGY OR EDUCATION SPECIAL OR ENGINEERING BIOMEDICAL OR COMPUTER SCIENCE INFORMATION SYSTEMS OR EDUCATION EDUCATIONAL RESEARCH OR VETERINARY SCIENCES OR INFORMATION SCIENCE LIBRARY SCIENCE OR COMPUTER SCIENCE THEORY METHODS OR COMPUTER SCIENCE INTERDISCIPLINARY APPLICATIONS OR ENGINEERING ELECTRICAL ELECTRONIC OR BIODIVERSITY CONSERVATION )  *Indexes=SCI-EXPANDED, SSCI, A&HCI, CPCI-S, CPCI-SSH, ESCI Timespan=1900-2021* |

Search Engine: Medline

| **#** | **Query** |
| --- | --- |
| 1 | ((Methodology checklist or scoring system or grading method* or scientific validity or clinical validity or appraisal tool or critical appraisal or grading quality or assessment or quality indicator* or evaluation or evaluating or methodological quality or grading or strength or framework) adj5 (clinical studies or preclinical studies or evidence or medical studies)).ab,kf,ti. |
| 2 | Evidence-Based Medicine/mt, st [Methods, Standards] |
| 3 | Evidence-Based Practice/mt, st [Methods, Standards] |
| 4 | Evaluation Studies as Topic/ |
| 5 | (evidence based standard* or evidence based method*).ab,kf,ti. |
| 6 | Models, Theoretical/ |
| 7 | Reference Standards/ |
| 8 | Methods/ |
| 9 | Quality Indicators, Health Care/ |
| 10 | (review* or systematic review* or meta-analysis).pt,ti. |
| 11 | 2 or 3 or 4 or 5 or 6 or 7 or 8 or 9 |
| 12 | 1 and 11 |
| 13 | 12 not 10 |
| 14 | limit 13 to ((english or french) and "humans only (removes records about animals)") |

Search engine: Embase

| **Query** |
| --- |
| (((('methodology checklist' OR 'scoring system' OR 'grading method*' OR 'scientific validity' OR 'clinical validity' OR 'appraisal tool' OR 'critical appraisal' OR 'grading quality' OR assessment OR 'quality indicator*' OR evaluation OR evaluating OR 'methodological quality' OR grading OR strength OR framework) NEAR/5 ('clinical studies' OR 'preclinical studies' OR evidence OR 'medical studies')):ab,ti AND (('evidence based standard*':ab,ti OR 'evidence based method*':ab,ti) OR ('framework'/de OR 'standard'/de OR 'evaluation study'/de OR 'procedures'/de OR 'quality indicator'/de OR 'scoring system'/de OR 'critical appraisal'/de OR 'assessment'/de))) NOT (review*:ti,it OR 'systematic review*':ti,it OR 'meta-analysis':ti,it)) AND 'human'/de AND ([english]/lim OR [french]/lim) |
